# Imported Malaria in Portugal: Prevalence of Polymorphisms in the Anti-Malarial Drug Resistance Genes *pfmdr1* and *pfk13*

**DOI:** 10.3390/microorganisms9102045

**Published:** 2021-09-28

**Authors:** Debora Serrano, Ana Santos-Reis, Clemente Silva, Ana Dias, Brigite Dias, Cristina Toscano, Cláudia Conceição, Teresa Baptista-Fernandes, Fatima Nogueira

**Affiliations:** 1 Global Health and Tropical Medicine, Instituto de Higiene e Medicina Tropical, Universidade NOVA de Lisboa (IHMT-NOVA), Rua da Junqueira 100, 1349-008 Lisboa, Portugal; a21001256@ihmt.unl.pt (D.S.); anareis@ihmt.unl.pt (A.S.-R.); a21000955@ihmt.unl.pt (C.S.); a21001219@ihmt.unl.pt (B.D.); claudiaconceicao@ihmt.unl.pt (C.C.); 2 Laboratório de Microbiologia Clínica e Biologia Molecular, Serviço de Patologia Clínica, Centro Hospitalar Lisboa Ocidental (CHLO), Rua da Junqueira 126, 1349-019 Lisboa, Portugal; apdias@chlo.min-saude.pt (A.D.); ctoscano@chlo.min-saude.pt (C.T.); tmfernandes@chlo.min-saude.pt (T.B.-F.)

**Keywords:** imported malaria, Portugal, CPLP, travel medicine

## Abstract

Malaria is one of the ‘big three’ killer infectious diseases, alongside tuberculosis and HIV. In non-endemic areas, malaria may occur in travelers who have recently been to or visited endemic regions. The number of imported malaria cases in Portugal has increased in recent years, mostly due to the close relationship with the community of Portuguese language countries. Samples were collected from malaria-infected patients attending Centro Hospitalar Lisboa Ocidental (CHLO) or the outpatient clinic of Instituto de Higiene e Medicina Tropical (IHMT-NOVA) between March 2014 and May 2021. Molecular characterization of *Plasmodium falciparum pfk13* and *pfmdr1* genes was performed. We analyzed 232 imported malaria cases. The majority (68.53%) of the patients came from Angola and only three patients travelled to a non-African country; one to Brazil and two to Indonesia. *P. falciparum* was diagnosed in 81.47% of the cases, *P. malariae* in 7.33%, *P. ovale* 6.47% and 1.72% carried *P. vivax*. No mutations were detected in *pfk13*. Regarding *pfmdr1*, the wild-type haplotype (N86/Y184/D1246) was also the most prevalent (64.71%) and N86/184F/D1246 was detected in 26.47% of the cases. The typical imported malaria case was middle-aged male, traveling from Angola, infected with *P. falciparum* carrying wild type *pfmdr1* and *pfk13*. Our study highlights the need for constant surveillance of malaria parasites imported into Portugal as an important pillar of public health.

## 1. Introduction

Malaria is transmitted through the bite of an infected female mosquito of the *Anopheles* genus. There are 5 species of malaria parasites that cause disease in humans: *Plasmodium falciparum*, *P. ovale*, *P. vivax*, *P. malariae* [1] and *P. knowlesi* [2]. In 2020, WHO registered 229 million malaria cases and approximately 0.5 million deaths in 87 endemic countries. More than 95% of cases and malaria-related deaths were caused by *P. falciparum* [3]. Once prevalent in almost every country in Europe, malaria transmission began to decline from the early 20th century and by 1975 Europe was declared free of endemic malaria transmission, with the exception of few restricted outbreaks related to the First and Second World Wars [4] and limited local outbreaks [3]. Nonetheless, France, Italy, Greece, Spain and Lithuania have recently recorded locally acquired malaria cases [5]. These cases resulted from transmission by local mosquitoes infected from imported malaria cases or by infected mosquitoes transported by aircraft from a malaria-endemic country (airport malaria) [5]. Malaria-receptive areas exist in Europe [6] due to the Mediterranean climate, allowing the presence of competent vectors [7]. In 2019, 8638 malaria cases were reported in the European Union/European Economic Area (EU/EEA). Among the 5509 cases with known importation status, nine were reported as acquired in the EU (two each by Germany, Greece, Spain and France, and one by The Netherlands) [5]. In 2020, Belgium also recorded two locally acquired cases near the airport [5].

In Portugal, before the 1950s, there were different levels of endemicity, which were largely associated with the suitability of habitat to the local vector, the mosquito *Anopheles atroparvus* [8,9]. By 1973, the indigenous strains of malaria parasites were considered eradicated from Portugal by the World Health Organization (WHO). *An. atroparvus*, currently circulating in Portugal, retains *competence* for transmitting imported strains of *P. falciparum* [10,11]. As the mosquito and the climatic conditions for transmission are still present in Portugal (and Europe), malaria re-emergence remains a real threat. In fact, locally acquired cases were documented in recent years in Italy and Greece [6,10,11,12,13]. Moreover, due to strong historical and cultural affinities, Portugal has a close relationship with numerous malaria-endemic countries, especially from Africa, namely the Países Africanos de lingua oficial Portuguesa (Portuguese-speaking African countries; PALOP) [14]. Travelers arriving from these regions provide a regular number of imported infections, ultimately contributing to the risk of autochthonous malaria transmission. In Portugal, an increasing number of *Plasmodium* infections have been reported by the Portuguese Ministry of Health during the last decade (from 54 cases in 2010 to 116 in 2019) [5]. From 2013 to 2019, the number of reported cases remained above 100 per year [5,15].

Before the Portuguese revolution of April 25, 1974, which brought democracy and independence to Angola, Mozambique, Guinea Bissau, São Tomé and Príncipe, Cape Verde (PALOP) and East Timor, many of the activities of research and training on “overseas exotic pathology” were localized at the Instituto de Higiene e Medicina Tropical, closely associated with the Overseas Hospital Egas Moniz (at present integrating Centro Hospitalar Lisboa Ocidental; CHLO). IHMT was integrated into the Nova University of Lisbon (becoming IHMT-NOVA) maintaining a travel clinic, and CHLO maintains ambulatory care at the infectious disease unit beyond emergency and inpatient care. These two units remain a national reference for tropical medicine.

The circulation of parasites between regions also increases the risk of importing drug resistance, which is underpinned by mutations in the *P. falciparum* genome. Anti-malarial drugs have long been important tools for malaria control [16]. However, *P. falciparum* has developed resistance to successively introduced antimalarials, including the currently used first-line treatment, the artemisinin-based combination therapies (ACTs; [17]). There are increasing reports on the persistence of *P. falciparum* parasitemia and elevated rates of reinfection following ACTs [18,19,20,21,22]. These suggest that the therapeutic efficacy of ACTs might have been compromised beyond Southeast Asia, where artemisinin resistance has been confirmed [23,24]. Approaches to the routine surveillance of antimalarial therapeutic efficacy include molecular testing of *P. falciparum* isolates from natural populations [25]. Multiple *P. falciparum* genes are involved in drug resistance.

The *P. falciparum* Kelch 13 (*pfk13*) gene is particularly important because single nucleotide polymorphisms (SNPs) on its propeller domain have been strongly associated with artemisinin resistance in Southeast Asia [26,27]. *P. falciparum* multidrug resistance gene 1 (*pfmdr1*; target for mefloquine, chloroquine and lumefantrine; [28,29]) is also implicated in *P. falciparum* tolerance to partner drugs of artemisinin derivatives (in ACTs). A selection of the *pfmdr1* N86/184F/D1246 haplotype has been reported from studies of artemether + lumefantrine (AL) treatment and recrudescence in patients in Asia and Africa [30,31,32,33].

Global vigilance against artemisinin resistance is vital for sustaining successes toward malaria elimination, particularly in Africa, where malaria is still a major health problem [24]. In this scenario, we conducted a retrospective survey to provide up-to-date analysis on the most relevant epidemiological characteristics and assessed the prevalence of mutations associated with drug resistance in *P. falciparum* parasites isolated from imported malaria cases since 2014 at the two above mentioned malaria reference facilities for tropical medicine in Portugal.

## 2. Materials and Methods

### 2.1. Ethic Statement

Anonymized data were collected retrospectively. The collection of patient data and processing and the analysis of parasite genes were performed under the IHMT Ethics Committee Approval 11.18 and 20.18 and CHLO Ethics Committee Approval RNEC:20170700050.

### 2.2. Data and Sample Collection

Malaria-infected patients diagnosed at IHMT (outpatient clinic) and at CHLO hospital (out and inpatient cases) in Lisbon, Portugal, between March 2014 and May 2021 were enrolled. The laboratory diagnosis of malaria was performed by rapid diagnostic tests and microscopy and later confirmed by PCR. All patients were first diagnosed by immune-chromatographic tests (rapid diagnostic tests RDTs) based either in malaria antigens *P. falciparum* histidine-rich protein 2 (PfHRP2) and the enzyme *Plasmodium* lactate dehydrogenase (pLDH), followed by the gold standard optical microscopic (OM) analysis of thick and thin blood smears (stained with Giemsa). OM enabled detection and identification of *Plasmodium* species as well as parasite load (parasites/μL of blood). Data referred to in Table 1 were taken from patient’s hospital records. Venous blood samples of malaria cases were collected for routine laboratory diagnostic procedures and the remaining after laboratory analysis were imbibed onto filter paper and stored at −20 °C for later molecular characterization of parasites. Parasite DNA was extracted using Chelex [34] and species confirmation by PCR [35].

### 2.3. Gene Sequencing and Analysis

Parasite samples with *P. falciparum* confirmed by PCR following the methodology previously published [35] were selected for sequencing of antimalarial drug resistance loci including: *pfmdr1* (PF3D7_0523000; including codons 86, 184 and 1246) and *pfk13* (PF3D7_1343700; including codons from 412 to codon 723). Genes were PCR amplified following the methodology previously published [34] with modifications (primers and thermocycling conditions in Table S1) and products cleaned using SureClean (Bioline, USA) following manufacturer’s instructions. The PCR products were analyzed by electrophoresis on a 2% agarose gel stained with GreenSafe (Nzytech, Portugal) to confirm amplification. The PCR products were sequenced using Sanger capillary platform at Eurofins Genomics, Germany, and the resulting sequences were analyzed using BLAST: Basic Local Alignment Search Tool (https://blast.ncbi.nlm.nih.gov/Blast.cgi; accessed on 18 August 2021). The laboratory-adapted 3D7 clone (MRA-102) was used as reference.

## 3. Results

### 3.1. Patients and Traveling History

**Figure 1:**
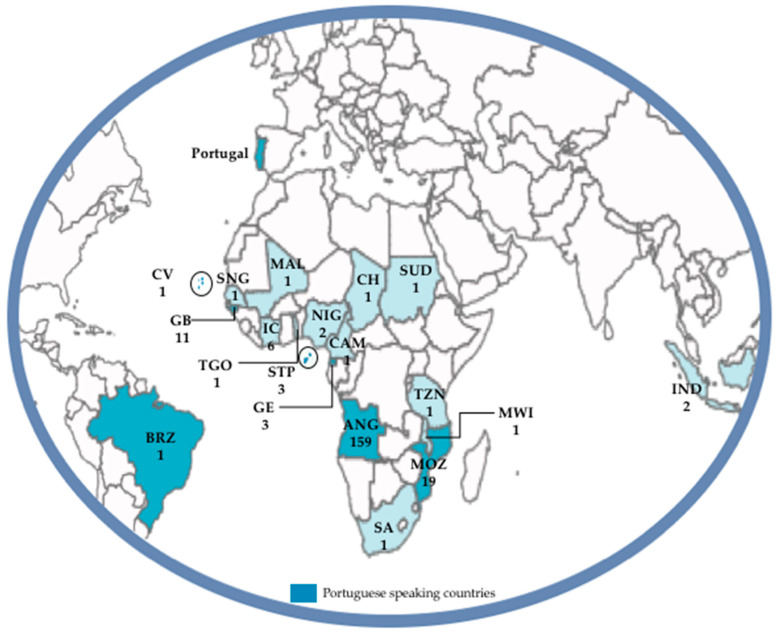
Geographical distribution of imported malaria cases. Parasite isolates’ country of origin is marked in blue. ANG, Angola; BRZ, Brazil; CV, Cape Verde; CH, Chad; CAM, Cameroon; GE, Equatorial Guinea; GB, Guinea Bissau; IND, Indonesia; IC, Côte D’Ivoire; MWI, Malawi; MAL, Mali; MOZ, Mozambique; NIG, Nigeria; STP, São Tomé and Príncipe; SNG, Senegal; SA, South Africa; SUD, Sudan; TZN, Tanzania; TGO, Togo.

Our study analyzed 232 patients who were diagnosed malaria positive at IHMT and CHLO laboratories from March 2014 to May 2021. The study identified 12 patients during 2014, 10 in 2015, 72 in 2016, 26 in 2017, 39 in 2018, 36 in 2019, 29 in 2020 and eight until the end of May 2021. The median age was 43 years (the youngest patient was five and the oldest 77 years) and the majority (72%; 167/232) were males. Sixteen of the 232 patients (6.90%) reported frequent travel to different African countries, hence were grouped as geographical provenance “Africa” in Table 1. The majority of the patients came from Angola (68.53%) followed by Mozambique (8.19%) and Guinea-Bissau (4.74%) (Table 1, Figure 1). Only three patients traveled to a non-African country; one to Brazil and two to Indonesia (Figure 1).

### 3.2. Laboratory Diagnosis

The isolates included in the present study were also analyzed by polymerase chain reaction (PCR) for *Plasmodium* species confirmation using 18S rRNA gene. No discrepancies were detected between RDTs, OM and PCR diagnostic results. Monoinfection with *P. falciparum* was detected in the majority of the parasite isolates (189, 81.47%), 17 (7.73%) were *P. malariae*, 15 (6.47%) *P. ovale* and only four patients (1.72%) carried *P. vivax*. Mixed infections were observed only in seven samples (Table 1). Interestingly, one of the patients arriving from Equatorial Guinea carried three of the five species that cause human malaria, *P. falciparum, P. ovale* and *P. vivax.*

### 3.3. Anti-Malarial Drug Resistance Associated Polymorphisms

**Figure 2:**
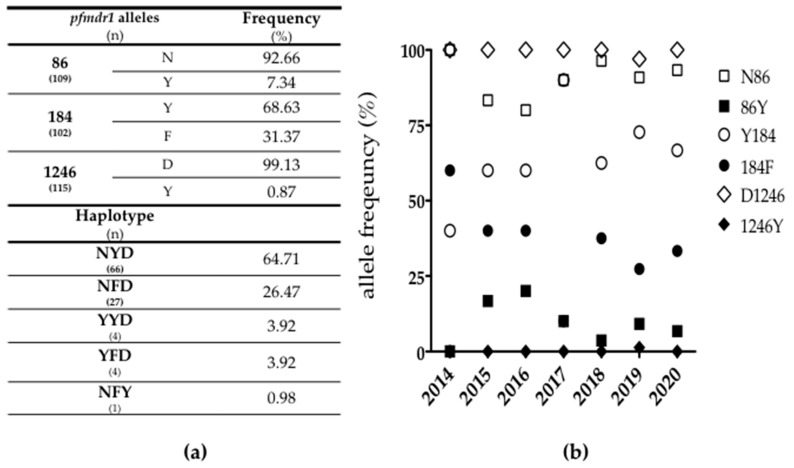
Molecular characterization of *P. falciparum pfmdr1* gene in imported parasites to Portugal. (**a**) *pfmdr1* allele and haplotype frequency; (**b**) Temporal trend in *pfmdr1* allele frequency, open symbols correspond to the wild type and filled symbols mutant alleles. The two samples from 2021 are not included in the graphic, one was NFD and for the other the positions 86 and 184 were not successfully sequenced and 1246 was wild type.

Of the 189 patients diagnosed with *P. falciparum* (single infections), we could only obtain a blood sample from 116 patients. Due to the retrospective nature of the study (regarding the years 2014, 2015 and 2016) we could not obtain parasite samples from all of these patients. This limits (somewhat) the estimation of the prevalence of the molecular markers for the correspondent years. Nevertheless, a total of 97 samples were successfully sequenced for *pfk13*, and all were wild type (identical to the reference, 3D7 clone MRA-102). None of the *pfk13* variants associated with artemisinin resistance in Southeast Asia [36] were observed in our study. A total of 109, 102 and 115 samples were successfully genotyped for *pfmdr1* codons 86, 184 and 1246, respectively. Mixed *pfmdr1* alleles were not observed (Table S2). Overall, wild-type alleles predominated with a frequency more than 90%, except for codon 184 where the wild-type allele (Y184) was found in 68.63% of the samples (Figure 2). Mutant alleles 86Y and 184F show a decreasing tendency over time (Figure 2), but these may not be significant due to the low number of samples in some of the years. On the other hand, the mutant allele 1246Y was only identified in one sample from Angola collected during 2019. Regarding *pfmdr1* haplotypes, the wild type (N86/Y184/D1246 or NYD) was the most prevalent with a frequency of 64.71%, followed by the haplotype associated with decreased susceptibility to AL (N86/184F/D1246 or NFD) with a frequency of 26.47% (Figure 2).

## 4. Discussion

In Portugal, malaria is a compulsory notification disease on the SINAVE (National Epidemiological Surveillance System) platform. Our study (a retrospective research study) analysed the imported malaria cases diagnosed with malaria at IHMT and CHLO laboratories from March 2014 to May 2021. IHMT and CHLO laboratories cases represent approximately 1/3 of the reported imported malaria cases in Portugal. Hence, our study represents only a fraction of the reported cases during the same period in Portugal nationwide [37]. However, there is evidence that the numbers of reported malaria [37,38] are underestimated. A study on imported malaria from 2000 to 2009 showed that reported malaria (based on mandatory, passive and comprehensive surveillance system, SINAVE) represented one-quarter of the hospital admissions (Diagnosis Related Groups (DRG) database of Portuguese mainland National Health Service hospitals) in the same period [39]. No marked seasonal trend was observed, although February, March, May and June were the months with a higher number of diagnosed cases. This does not coincide with the reported overall tendency for the EU countries [37], where the cases increase during and immediately after the summer holiday months (July–September). Most of the imported malaria cases to non-endemic countries are linked to travel routes from historical, economic, linguistic and cultural links with endemic areas [40], and in the case of Portugal particularly from Africa [14,40,41,42]. The reasons for the traveling were not the object of inquiry in our study, hence we cannot make a definitive correlation between the number of cases and a particular traveling motive. Nonetheless, a recent study on travel-acquired health problems in Portugal indicated that 78.2% occurred in the contexts of business/employment and volunteering and only 36.9% in the context of tourism [42]. Portugal has a large resident community from the PALOPs and Brazil (a total of 290.292; [43]) who receive and travel to visit friends and relatives from malaria-endemic home countries and probably make up more than 3% of travelers to and from these destinations [42]. These might contribute to the year-round imported malaria cases, rather than being concentrated immediately after the summer holiday months [37,40]. Malaria cases were mostly men (approximately 72%), middle-aged (median 43) and traveling from Angola, in line with previous studies [14,39,41,42]. The reason why the vast majority of patients were travelers from Angola is probably linked to business, after the 2008 Portuguese economic crises [44,45].

We found a dominance of *P. falciparum* in 81.47% of the cases from Africa, including five mixed infections (Table 1) as compared with those originating from other regions (Brazil 0.46% and Indonesia 0.92%). This dominance reflects probably: (i) the geographic distributions of *P. falciparum*, which is more prevalent in Africa [1,3,40]; (ii) the endemicity of malaria in the regions of origin, Africa accounting for more than 90% of world malaria cases during the last decades [3]; and (iii) the fact that the majority of travel in Portugal for endemic regions, were to Africa [37,39,42].

*P. malariae* was the second most represented, with a prevalence (7.33%) within the expected range. It is widely distributed in sub-Saharan Africa with overlapping presence with *P. falciparum* [46,47]. *P. ovale*, also endemic in Africa, where it probably represents the main agent of relapsing malaria, had a prevalence of 6.47% falling within the reported range [48,49,50,51,52]. *P. vivax* was identified in mixed infections with *P. falciparum* (three cases) and as a single infection (one case) in patients arriving from Africa. This was not a surprise since *P. vivax* has been reported as endemic in Africa [53,54], even infecting Duffy-negative red blood cells [55,56]. Non-falciparum malaria represented (≈25%) of imported malaria cases in our study, which is in line with imported malarias diagnosed in Europe [5,37].

The information on imported malaria into Portugal is scarce, not systematized and mostly available through individual manuscripts, reflecting (probably) part of the total imported cases into the country, as is the case with the present work. To the best of our knowledge, the present work is the first to present a description of the drug-resistance associated genotypic background of a sizable imported parasite population into Portugal.

In Southeast Asia, the correlation of specific SNPs in the gene *pfk13* with delayed parasite clearance (decreased parasite susceptibility to ACTs), has generated molecular markers for the surveillance of ACT-resistant parasites [26,36,57]. Mutations in the *pfk13* gene were not detected among the *P. falciparum* isolates in our study. The *pfk13* SNPs, previously shown to be associated with slow artemisinin clearance (positivity on day 3) of *P. falciparum* in Southeast Asia, have rarely been reported in Africa [58,59,60]. Nevertheless, nine of these SNPs (C469Y, R515K, S522C, V568G, P574L, A675V, F446Ile, Met476I and P553L) have already been detected, mainly in east Africa [61]. Other non-synonymous mutations have also been identified here (although lacking drug response association) and there is still little evidence of ACT-resistant parasites circulating in Africa [60,61]. Due to their regional genetic background, African parasites might select different SNPs from those in Southeast Asia [62] or other genes might be involved as well [63,64,65].

The presence of SNPs or haplotypes linked to tolerance of artemisinin derivatives can impact parasite survival, potentially resulting in persistence of symptoms and an increased local parasite reservoir. We report a moderate frequency of the *pfmdr1* NFD haplotype (26.47%) and a high prevalence of NYD (64.71%), which are associated with parasite reduced susceptibility to AL [66]. Prevalence of NFD and NYD haplotypes follow recently published data from Angola [67,68] (the country of origin of the majority of our samples), supporting the worrisome conjecture that a substantial part of the imported malaria cases in Portugal carry genotypes associated with reduced susceptibility to (at least) AL. We also detected a high frequency of wild-type alleles at codons 86 and 1246 (92.66% for N86 and 99.13% for D1246). *Pfmdr1* mutant allele 86Y is associated with a longer time to reinfection after AL treatment [69], reinforcing its informative value as a molecular marker of antimalarial drug susceptibility. *Pfmdr1* 86Y has been decreasing in many parts of Africa where AL is used, which is consistent with the expected direction of selection [69,70,71]. Frequency of N86 and D1246 in our isolates, follow the tendency reported from Africa [71,72,73,74] and in particular from Angola [67,68,75] coinciding with the geographical origin of our samples.

In 1975, the WHO announced that malaria had been eradicated in Europe and all recorded cases since were introduced through migration [37,76,77]. European countries carry the highest global burden of imported malaria cases in absolute numbers and relative to the population size of each country [78]. Currently, nearly all malaria cases reported by EU countries are imported [37,40] but there have been local transited cases in Italy, Greece, Spain, Germany, UK and France [15,79,80,81,82]. Outbreaks rarely occur, despite significant numbers of imported malaria cases [37,40,83]. Southern Europe is among the most risky regions for malaria resurgence [84,85] due to local environmental features such as temperature, humidity, land use or vector accessibility to humans, which represent factors strongly influencing the efficiency of anopheline mosquitoes to transmit malaria [7,86,87,88,89]. The dominant *Anopheles* vector species in Europe and in Portugal is currently *An. atroparvus* [87,90,91]. When malaria was endemic in Portugal, *An. atroparvus* was the most efficient vector [9,92]. There is already a risk of malaria transmission in Portugal, and current environmental conditions are suitable for the vectors’ development [9,10,87,89]; in fact, the distribution of the vector is similar to the period before malaria elimination [9,87].

Previous studies revealed that *An. atroparvus* was probably not susceptible to the African *P. falciparum* strains [86,88,90,91,92,93], but susceptible to the European strains [88,93,94], although probably fully susceptible to infection by *P. vivax* strains imported from Africa [80]. Hence, as reflected by the detection of autochthonous cases in European countries [15,79,80,81,82], a transmission resurgence risk in Europe should be taken into account.

In Portugal, there is a clear need for a systematic and more efficient method of early diagnosis, monitoring and referral of malaria cases to an appropriate health facility, not only to reduce probability of reintroduction [95] but to prevent severe disease and death. Recently, two studies involving 59 and 98 patients reported case fatality of 15.2% [14] and 4.1% [96], respectively. Malaria is a curable disease. Prompt and accurate diagnosis is decisive to effective disease management [97].

## 5. Conclusions

The typical imported malaria case was middle-aged male, traveling from Angola infected with *P. falciparum* carrying wild type *pfmdr1* and *pfk13*. The boost in recent years of people circulation between Portugal and malaria-endemic regions, increases the risk of reintroducing drug-resistant malaria parasites into Portugal. These make the constant monitoring of the imported malaria parasites to Portugal an imperative and important pillar of public health. Furthermore, it is necessary to maintain surveillance of the emergence and spread of mutations in genes associated with response to ACTs, such as *pfk13* and *pfmdr1*, because the geographic dispersion of drug-resistant malaria parasites can cause devastating effects.

## Figures and Tables

**Table 1:** Malaria imported cases diagnosed at IHMT and CHLO from 2014–2021 and geographical origin.

Malaria Cases (*n* = 232)		
Country of Origin *n* (%)		Species (*n*)
**Angola**	159 (68.53)	Pf 133; Pm 15; Po 9; Po/Pm 2;
Cameroon	1 (0.43)	Pf 1
**Cape Verde**	1 (0.43)	Pf 1
Chad	1 (0.43)	Po 1
Côte D’Ivoire	6 (2.59)	Pf 6
Equatorial Guinea	3 (1.30)	Pf 2; Pf/Pv/Po 1
**Guinea Bissau**	11 (4.74)	Pf 11
Malawi	1 (0.43)	Pf 1
Mali	1 (0.43)	Po 1
**Mozambique**	19 (8.19)	Pf 15; Po 3; Pf/Po 1
Nigeria	2 (0.86)	Pf 1
**São Tomé and Príncipe**	3 (1.30)	Pf 3
Senegal	1 (0.43)	Pf/Pv 1
South Africa	1 (0.43)	Pf 1
Sudan	1 (0.43)	Pf 1
Tanzania	1 (0.43)	Pf 1
Togo	1 (0.43)	Pf 1
Africa *	16 (6.90)	Pf 10; Pv 1; Pm 2; Po 1; Pf/Pv 1; Pf/Pm 1
Brazil	1 (0.43)	Pv 1
Indonesia	2 (0.86)	Pv 2

% was calculated relatively to the total of the 232 samples; highlighted in bold Portuguese speaking countries; * frequent travelers from and between more than one African country; Pf, *P. falciparum*; Po, *P. ovale*; Pv, *P. vivax*; Pm, *P. malariae*.

## Data Availability

All reported data is provided in the body of the text and in the Supplementary Materials Tables S1 and S2.

## Supplementary Materials

The following are available online at https://www.mdpi.com/article/10.3390/microorganisms9102045/s1, Table S1: Primers and thermocycling conditions for PCR amplification of *pfmdr1* and *pfk13* gene fragments; Table S2: Molecular characterization of *P. falciparum* isolates.
