# Supplementary material for: Imported Malaria in Portugal: Prevalence of Polymorphisms in the Anti-Malarial Drug Resistance Genes pfmdr1 and pfk13
Source: Microorganisms. 2021 Sep 28;9(10):2045. doi: 10.3390/microorganisms9102045 (PMC8538333; doi:10.3390/microorganisms9102045)
Supplement: Supplementary file 1 [file microorganisms-09-02045-s001.zip › microorganisms-1385552-supplementary.pdf]

Table S1. Primers and thermocycling conditions for PCR amplification of *pfmdr1* and *pfk13* gene fragments.

Gene	Primer	Primer (5′-3′)′	PCR conditions
pfmdr1	86/184F	ATGGGTAAAGAGCGAAAGAG	94 °C for 2 min; 10 × [94 °C for 1 min; 60 °C for 30 sec; 72°C for 1 min.]; 72 °C for 3 min.
	86/184R	GTCTTTTCTCCACAATAACTTGC	
	86/184F2	GTATGTGCTGTATTATCAGGAGGA	
	86/184F3	AACAGTTCTTATTCCCATTAAAGCC	
	1246F	CTACAGCAATCGTTGGAGAA	
	1246R	GAGAATAGCTATAGCTAGAGC	
pfk13	k13F	GAAATCCGTTAACTATACCC	94 °C for 2 min; 10 × [94 °C for 1 min; 57 °C for 30 sec; 72 °C for 1 min.]; 72 °C for 3 min.
	k13R	GGAGTGACCAAATCTGGGA	
	13NF2	GAGATGTATGGTATGTTTCAAG	

Table S2. Molecular characterization of *P. falciparum* isolates.

	pfmdr1 allele frequency						
	**N86Y**		**Y184F**		**D1246Y**		
**Year**	**N**<br>***n*; %**	**Y**<br>***n*; %**	**Y**<br>***n*; %**	**F**<br>***n*; %**	**D**<br>***n*; %**	**Y**<br>***n*; %**	**Haplotype**<br>***n*; %**
2014	6; 100	0	2; 40	3; 60	6; 100	0	NYD 2; 40<br>NFD 3; 60
2015	5; 83.3	1; 16.7	3; 60	2; 40	6; 100	0	NYD 2; 40<br>NFD 2; 40<br>YYD 1; 20
2016	4; 80	1; 20	3; 60	2; 40	5; 100	0	NYD 3; 60<br>NFD 1; 20<br>YFD 1; 20
2017	9; 90	1; 10	9; 90	1; 10	12; 100	0	NYD 8; 80<br>NFD 1; 10<br>YYD 1; 10
2018	27; 96.4	1; 3.6	15; 62.5	9; 37.5	29; 100	0	NYD 15; 62.5<br>NFD 8; 33.3<br>YFD 1; 4.2
2019	30; 90.9	3; 9.1	24; 72.7	9; 27.3	32; 97	1; 3	NYD 22; 66.7<br>NFD 7; 21.2<br>NFY 1; 3<br>YYD 2; 6.1<br>YFD 1; 3
2020	14; 93.3	1; 6.7	10; 66.7	5; 33.3	18; 100	0	NYD 10; 66.7<br>NFD 4; 26.7<br>YFD 1; 6.6
2021	6; 100	0	4; 80	1; 20	6; 100	0	NYD 4; 80<br>NFD 1; 20
